# Editorial: Excessive and problematic smartphone usage

**DOI:** 10.3389/fpsyt.2022.972613

**Published:** 2022-07-20

**Authors:** Aviv Weinstein, Kristiana Siste

**Affiliations:** ^1^Department of Psychology and Behavioral Science, The Isadore and Ruth Kastin Chair for Brain Research University of Ariel, Ariel, Israel; ^2^Department of Psychiatry, Faculty of Medicine, Universitas Indonesia, Depok, Indonesia

**Keywords:** problematic smartphone use, excessive smartphone use, social media addiction, problematic smartphone and internet usage, social media

The excessive use of computer screens and smartphones is raising serious concerns among health and educational authorities due to the adverse effects of such use on children and adolescents. During COVID-19, internet usage is increasing. This is supported by the mobility of the gadgets used, one of which is a smartphone. Smartphones have been designed to be more sophisticated over the years, thus increasing a person's motivation and ease in using the internet. Smartphones can be used for various purposes, such as playing games, socializing using social media, shopping for daily necessities, and others. As a result of the convenience provided by the smartphone, someone could use this gadget continuously. Excessive smartphone use requires special attention because excessive use can lead to addiction and affect all aspects of a person's life. Twelve papers have examined various psychological, behavioral, and health issues associated with problematic smartphone use in this Research Topic.

An overall review of the literature by Wacks and Weinstein has shown that excessive smartphone use is associated with psychiatric, cognitive, emotional, medical, and brain changes that should be considered by health and education professionals. Studies showed comorbidity of excessive smartphone use with depression, anxiety, OCD, ADHD, and alcohol use disorder. Excessive smartphone use was also associated with difficulties in cognitive-emotion regulation, impulsivity, impaired cognitive function, addiction to social networking, shyness, and low self-esteem. Medical problems included sleep problems, reduced physical fitness, unhealthy eating habits, pain and migraines, reduced cognitive control, and changes in the brain's gray matter volume. Pera has found that depression and social anxiety constitute risk determinants for greater problematic smartphone use and that particular categories of smartphone applications are positively related to wellbeing.

Furthermore, state anxiety and motivations are significant predictors of problematic smartphone use and affect participation in social engagement. Eichenberg et al. have found that respondents with problematic smartphone use showed higher scores of extraversion and neuroticism and higher rates of depression and anxiety. Contrary to expectations, they showed higher values for perceived social support than individuals without problematic smartphone use. They have argued that personality traits should be considered during therapy for problematic smartphone use.The neurobiology of excessive smartphone use has been examined in two brain imaging studies. Paik et al. have investigated resting-state functional connectivity (rsFC) of the insula, which is implicated in salience processing, interoceptive processing, and cognitive control in adults who used smartphones by functional magnetic resonance imaging (fMRI). They hypothesized that prolonged bedtime smartphone use might serve as one of the relevant behavioral measures of problematic smartphone use, and prolonged bedtime smartphone use might also be associated with insula-centered functional connectivity. Insula has been known to control the human brain's cognitive function, homeostatic, and affective processes. It can influence decision-making processes and integrate internal and external physiological signs in uncertain situations. They have found that prolonged bedtime smartphone use was associated with higher smartphone addiction proneness scale but not with sleep quality. The findings imply that prolonged bedtime smartphone use can be an essential behavioral measure of problematic smartphone use, and altered insula-centered functional connectivity may be associated with it. This study shows that there is currently more effort in describing the PSU phenomenon because there is no standardized consensus on PSU.

Ahn et al. have performed a resting state seed-based functional connectivity (rsFC) analysis in problematic smartphone users and found enhanced FC within the salience network and between the salience and default mode network. The salience network, consisting of nodes in the right frontoinsular cortex and anterior cingulate cortex, works in the process of orientation to stimuli and allocation of attention. Meanwhile, the central executive network consisting of the dorsolateral prefrontal cortex and the posterior parietal cortex has a function in the decision-making process. The default mode network which consists of the medial prefrontal cortex, the rostral part of the anterior cingulate cortex, the precuneus, and the posterior cingulate cortex works in thought processes. Finally, the affective network is responsible for regulating and perceiving emotions and is associated with structures in the amygdala and anterior cingulate subgenual cortex. Moreover, there was decreased FC between the salience and central executive network in problematic smartphone users. They argued that problematic smartphone use is associated with changes in FC of key salience networks. Overall, this study shows neural changes due to smartphone addiction in terms of salience, central executive, default mode, and affective networks.

Olson et al. have found a positive correlation between hypnotisability and smartphone addiction. They have suggested that targeting the absorbed, time-distorted, and automatic use of smartphones may promote healthier phone habits. Gao et al. have found associations between behavioral inhibition/activation systems (BIS/BAS) and problematic mobile use. They have found a positive association between BIS and “mood modification” or “tolerance” and between BAS-fun seeking and “mood modification” or “conflict.” These findings explain how individuals with a high behavioral inhibition or activation system may develop problematic mobile use. The results of this study have implications for prevention programs to overcome problematic mobile phone use.

The relationships between excessive smartphone and social media use and the COVID-19 pandemic were examined in two studies. The impact of excessive smartphone and social media use during the COVID-19 pandemic was examined among Bangladeshi college and university students by Islam et al. Excessive smartphone and social media use were associated with lower age, poor sleep, social media use, watching television, anxiety, and depression. Additionally, problematic social media use was associated with being female, living with nuclear family, having urban residence, irregular physical exercise, poor engagement with academic studies, and avoiding earning activities, whilst being male, being married, living with lower-income family, and alcohol consumption were associated with problematic social media use. Zhang et al. examined the relationship between problematic smartphone use, sleep quality, and daytime fatigue among medical students during COVID-19 in six polyclinic hospitals in Beijing. The COVID-19 pandemic has caused medical students to learn primarily online, thereby increasing the risk of problematic smartphone use (PSU) and various other negative impacts such as psychological symptoms. The study used a Short Version Smartphone Addiction Scale (SAS-SV) questionnaire and found that 49.7% of medical students had PSU. Participants with problematic smartphone use reported sleep disturbance, physical fatigue, and mental fatigue. Sleep quality mediated the relationship between problematic smartphone use and daytime fatigue.

Two studies used questionnaires to assess the intention to use smartphones and the risk of smartphone distraction. Choi et al. have investigated the users' behavioral intention to use smartphone management applications in South Korea. They have found that in both non-problematic smartphone use groups and problematic smartphone use groups, facilitating factors and perceived security positively affected users' intentions to use the application. Zhao et al. evaluated the Chinese version of the Smartphone Distraction Scale (C-SDS) to screen the risk of smartphone distraction in Chinese college students. They have shown a 3-factor structure, which consisted of attention impulsiveness, multitasking, and emotion regulation. Females, the purpose of using a smartphone, smartphone usage, fear of missing out, smartphone addiction, and positive and negative metacognitions about smartphone use were related to the C-SDS. C-SDS was found to be valid and reliable among Chinese college students. Zhou and Wang have used a self–reported questionnaire to examine the effects of aerobic exercise and reading on inhibitory control in college students with excessive mobile use. They have used the anti-saccade task to examine the differences in the effects of aerobic exercise and reading on inhibitory control of college students with mobile phone addiction. They have shown that although exercise and reading affect inhibitory control of college students with excessive smartphone use, the effect of reading may be somehow superior to exercise. The questionnaire regarding smartphone addiction needs to be investigated further to produce a standard in terms of diagnosis. Further research is needed in order to produce a sensitive and specific measuring tool for establishing the diagnosis of smartphone addiction. Smartphone users have clinical characteristics similar to other behavioral addiction disorders such as internet gaming disorder and gambling disorder. Excessive use of smartphones has symptoms of the behavior of loss of control and the continuation of smartphone use despite negative consequences. In addition, excessive smartphone use is also reported to have a negative impact on daily activities at work and socially. Based on the Interaction of Person-Affect-Cognition-Execution model, there is a relationship between the imbalance in craving/cues—reactivity with inhibitory control that causes a person to experience behavioral addiction. Smartphone use is also associated with the same neurobiological and cognitive problems as other behavioral addictions, thus creating a conceptual framework where smartphone addiction might be included in the diagnostic criteria in the DSM or ICD. Until now, there is no consensus regarding the pathway and criteria for diagnosing smartphone addiction.

In conclusion, we have collected studies showing that problematic smartphone use was closely related to disturbances in cognitive-emotional regulation, impulsivity, impaired cognitive function, addiction to social networks, shyness, low self-esteem, depression, anxiety, and higher scores of extraversion and neuroticism. PSU is also linked to medical problems, including sleep problems, decreased physical fitness, unhealthy eating habits, pain, migraines, decreased cognitive control, brain gray matter volume changes, and decreased FC between the salience and central executive network. These have become important during the COVID-19 pandemic. Several questionnaires have been developed to assess the risks of having problematic smartphone use. Treatment studies are urgently needed.

## Author contributions

AW and KS have contributed to the writing of this editorial. Both authors contributed to the article and approved the submitted version.

## Conflict of interest

The authors declare that the research was conducted in the absence of any commercial or financial relationships that could be construed as a potential conflict of interest.

## Publisher's note

All claims expressed in this article are solely those of the authors and do not necessarily represent those of their affiliated organizations, or those of the publisher, the editors and the reviewers. Any product that may be evaluated in this article, or claim that may be made by its manufacturer, is not guaranteed or endorsed by the publisher.

